# Final-year medical students’ reflections on types of significant events in primary care

**DOI:** 10.4102/phcfm.v15i1.4099

**Published:** 2023-11-01

**Authors:** Samantha Dube, Motlatso Mlambo, Nontsikelelo O. Mapukata

**Affiliations:** 1 Department of Family Medicine and Primary Care, Faculty of Health Sciences, University of the Witwatersrand, Johannesburg, South Africa; 2 Department of Institutional Intelligence, Portfolio: Strategy, Risk and Advisory Service, University of South Africa, Johannesburg, South Africa; 3 Division of Public Health Medicine, Faculty of Health Sciences, University of Cape Town, Cape Town, South Africa

**Keywords:** adverse events, medical students, patient safety, primary health care settings, significant events analysis

## Abstract

**Background:**

Adverse events are considered a universal challenge and a burden in the provision of healthcare. For that reason, significant event analysis (SEA) is a critical undertaking in primary health care (PHC), particularly in South Africa where 84% of the population relies on the public health system for their care.

**Aim:**

The study aimed to describe the types of perceived significant events medical students experienced during an integrated primary care block placement.

**Setting:**

Eighteen PHC settings included clinics, community health centres and district hospitals across three provinces in Gauteng, Mpumalanga and the North West.

**Methods:**

Using a qualitative descriptive design with purposeful sampling and maximum variation, structured reflection reports were retrieved from logbooks of final-year medical students studying at a South African university in 2014. Conventional content analysis was used to record the relevant facets of secondary data from 124 logbooks that contained a recording of a significant event using MAXQDA software version 2020.4.

**Results:**

An iterative process revealed three major themes of significant events that were prevalent in PHC settings. These comprised medication and prescription errors, diagnostic errors and suboptimal patient management.

**Conclusion:**

Significant event analysis became a critical quality improvement reflective learning tool. Logbooks offered an opportunity for medical students to explore significant events as a strategic way towards addressing quality and safe practices in PHC settings.

**Contribution:**

This study demonstrated medical students’ ability to identify incidents in the care of patients using the SEA approach and their role in assessing patient safety issues in PHC settings.

## Introduction

Adverse events in medical care present a challenge in the provision of public healthcare as this experience tends to compromise patient safety (Table 1). Accordingly, Slawomirsk and colleagues reported that for every 10 patients presenting in outpatient and primary health care (PHC) facilities worldwide, four experience harm.^1^ Patient harm is recorded as the 14th determinant of the global burden of disease compared to malaria and tuberculosis.^2^ With about 421 million people hospitalised annually, one-tenth of patients reportedly experience adverse events globally.^3^ Annually, hospitals in low- and middle-income countries experience 134m adverse events resulting from poor patient care.^4^ Despite these recorded events, there is insufficient information on the safety of patients in primary care settings in comparison with secondary care.^5,6^ In the United Kingdom alone, it is estimated that 2% of consultations in primary care result in adverse events.^7^ In sub-Saharan Africa, adverse events are outcomes of healthcare provision in impoverished communities where there are high levels of illiteracy. Coupled with that, late hospital presentations and insufficient infrastructure, patients become susceptible to significant events despite the efforts of healthcare professionals to provide quality healthcare.^8^ Such conditions bring to the fore the public health burden of significant events and the potential threat they pose to patient safety.^9,10^

**TABLE 1:** Definition of terms.

Term	Definition
Adverse event	An event that results in unintended harm to the patient and is related to the care and/or services provided to the patient rather than to the patient’s underlying medical condition.^18,19,20^
Disclosure	The process by which an adverse event is communicated to the patient by healthcare workers.^21,22^
Error (medical)	An act (plan, decision, choice, action or inaction) in patient care that, when reviewed, was not correct and resulted in patient harm or a near miss.^23^
Patient safety	The pursuit of reduction and mitigation of unsafe acts within the healthcare system, as well as the use of best practices shown to lead to optimal patient outcomes.^24^
Primary health care	The first point of contact people has with the healthcare system which provides comprehensive, accessible, community-based care that meets the health needs of individuals throughout their life.^25^
Significant event	Any event deemed significant by a member of a healthcare team in the care of a patient and the way in which this care was provided.^14^
Significant event analysis	A team safety investigation and quality improvement tool used to help understand the event and why it happened and direct subsequent learning and improvement efforts.^26^

Note: Please see the full reference list for more information.

This maldistribution and burden of disease in South Africa emphasise the need for a health system to deliver quality health services to the people who need them the most.^11^ Structural inequities and inequitable resource allocation in health service delivery highlight the disparities in rural areas, underserved communities and urban areas.^12^ These structural inefficiencies tend to compromise the quality of care, leaving PHC facilities to deal with the consequences of these inequalities. Taking into consideration that 84% of the South African population rely on PHC services that are offered in public health facilities,^13^ significant event analysis (SEA) provides a platform to learn from reported errors in order to offer quality patient care. However, it is challenging to do so at present as there is limited information on patient safety threats in primary care and their solutions, despite the quantification of incidences of error in secondary care.^14^

Medical students’ involvement in SEA provides insights into approaches to managing errors in practice.^15^ However, medical students are often uncomfortable disclosing adverse events they have committed or witnessed.^16^ This is because there is a lack of role models in medical education who readily admit to committing an error, thus imparting the principle of accountability and commitment to patient safety.^15^ Failure to disclose a significant event presents a missed teaching opportunity for medical students to learn about managing adverse events.^17^ It also hinders healthcare facilities from improving the quality of care provided, as they would not be aware of the causes and factors associated with the significant events.

To our knowledge, there is no documented literature yet on medical students’ experiences of a significant event in South Africa’s PHC settings. As such, the aim of this article is to report on findings from a study undertaken as part of a master’s degree in public health that explored medical students’ reflections on perceived significant events during their Integrated Primary Care (IPC) block placement in PHC settings. This article describes the types of significant events experienced by medical students studying at a South African university.

## Research methods and design

### Study design

This was a secondary data analysis study based on a section of the IPC block logbook focusing on medical student reflections on a significant event. A qualitative descriptive research design was used to portray the significant events in PHC facilities.^27,28,29^ This design was suitable for secondary data analysis, which described the types of significant events medical students were involved in and factors associated with the significant events.^30^ The value of qualitative description was therefore not only in the knowledge generated but also in determining significant, important findings and their implications.^31^

### Study setting and participants

**FIGURE 1:**
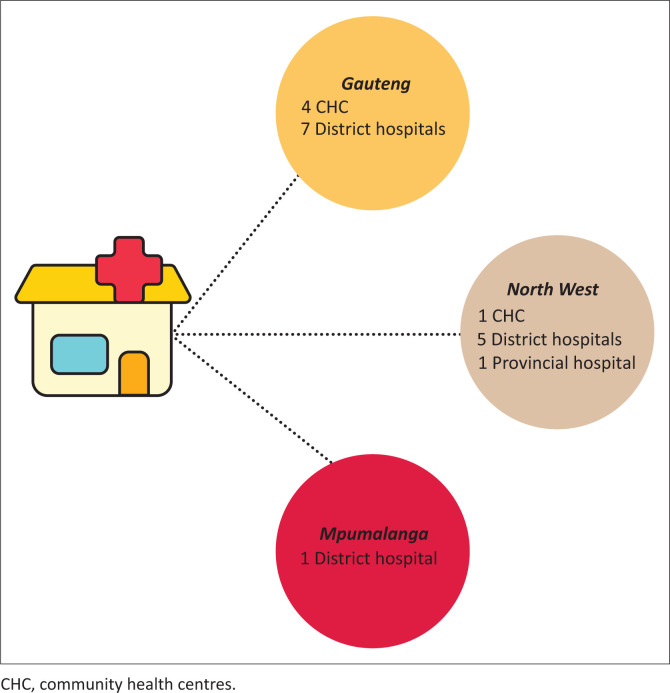
Study sites.

In 2014, participants in this study were final-year Wits medical students based in 18 rural and urban healthcare facilities in Gauteng, Mpumalanga and North West provinces (Figure 1) as part of their IPC block 6-week placement. Primary healthcare facilities included district hospitals and community health centres (CHCs) with their supporting clinics in all three provinces.^32^

### Data collection

As part of the IPC block, 228 medical students documented in their logbooks reflections on exposure to different aspects of PHC. Each student was required to complete a pre-structured reflective diary with six open-ended questions about a significant event they had either ‘witnessed or were involved in at their site during the 6-week clinical rotation where management of the patient was sub-optimal, an adverse event occurred, or a patient died’. Although this formed part of the logbook, like portfolios,^33^ entries about adverse events were a critical reflection tool that facilitated students in learning about the management of significant events in PHC settings. Maximum variation purposeful sampling allowed the selection of participants directly involved in the significant event they recorded (*n* = 124) and eliminated those who described significant events they had observed from a distance or overheard the event discussed by colleagues.^34,35^

### Data analysis

In preparing for analysis, secondary data were exported to a Microsoft Word document; its quality was assessed and organised to ensure that it aligned with the aims of the study.^36^ With a view to accurately record the significant facets of the data related to the research questions, conventional content analysis was used.^37^ Data were coded using MAXQDA software version 2020.4.

### Ethical considerations

Ethical clearance to conduct this study was obtained from the University of the Witwatersrand Human Research Ethics Committee (Medical) (No. M170853). Written informed consent was obtained from all final-year medical students prior to participation in the study allowing the Wits Centre for Rural Health to use their logbook reflections during the IPC rotation for research purposes. To ensure confidentiality, no names were collected from the logbooks and data were kept in a password-protected laptop. The research team did not have access to the participant’s personal information.

## Results

The analysis revealed three major themes that outlined the types of significant events witnessed during clinical rotations, namely medication and prescription errors, patient diagnosis errors and suboptimal patient management. Many of the perceived significant events were reported in the emergency medicine rooms and in the maternity and labor wards of the PHC facilities where participants were based for their IPC block.

### Theme one: Medication and prescription errors

For these perceived significant events, participants recorded incidences where patients received wrong medication or instances where there were prescription errors. Some participants reported that patients received an incorrect treatment regimen for the condition they presented with:

‘A patient was diagnosed with MDR-TB, but he was put on first-line regimen regardless. After 6/12 he was obviously not better, so after investigating, the sisters in the TB clinic at [*name of facility withheld*] picked up the mistake in his management.’ (Participant 7, Rotation 3, Facility 2)

Participants indicated that some healthcare workers did not take medication contraindications into account. This highlighted the poor prescribing habits of some healthcare workers, especially for patients with chronic conditions:

‘Although many such incidents occurred like this, it will describe the poor prescribing habits of some doctors in the chronic clinic … a patient with severe gout being dosed on allopurinol and put on Hydrochlorothiazide [*HCTZ*] for two years.’ (Participant 77, Rotation, 1, Facility 2)

Patient identity mix-ups were a common occurrence, especially where patients had similar names. Consequently, patients got the wrong medication that was not necessarily prescribed for their condition:

‘Patients with the same names were attending chronic patient clinic. Both had hypertension, but only the one had schizophrenia as a co-morbidity. When finding the EDL packets, the psych patient’s injection was given to the HPT patient wrongly. Patient did not think she was wrongly being medicated.’ (Participant 34, Rotation 4, Facility 7)

### Theme two: Patient diagnosis errors

Some participants highlighted wrong patient diagnosis and missed diagnosis as areas of concern that resulted in perceived significant events during their clinical rotations.

Disagreements among healthcare workers on patient diagnosis and course of treatment were perceived as significant events, which resulted in suboptimal care of the patient:

‘A patient came in with a snake bite rumoured to be a black mamba bite. Doctor in charge delayed care as they weren’t certain if it was a black mamba and the patient deteriorated. Nurse disagreed with the treatment.’ (Participant 29, Rotation 5, Facility 14)

Some participants perceived that their suggestions for the diagnosis and management of patients were not taken into account as they were students:

‘A patient presented during my mini-CEX with the doctor at the clinic with a history of persistent cough and occasional blood stains and night sweats. He had a contact with a similar problem. I was concerned that he had TB, but the doctor believed he had interstitial lung disease due to the fact that he worked in the mines. He was sent home with Prednisone, Beclomethasone and Asthavent.’ (Participant 46, Rotation 4, Facility 11)

This could have resulted in poor patient care as students could not contradict and voice their own opinions against their seniors.

### Theme three: Suboptimal patient management

Healthcare workers’ attitudes, poor communication, poor management of assault cases and labour cases all resulted in poor patient care. Health workers’ attitudes towards patients, in the form of physical and verbal abuse, were ongoing concerns:

‘… When trying to lift the patient off the wheelchair onto the bed, the nurse got irritated that the patient was not carrying her own weight and began hitting her and pulling at the patient’s clothes and shouting at her.’ (Participant 28, Rotation 5, Facility 3)

Some participants recorded poor management of physical and sexual assault cases as significant events that affected patients’ access to quality healthcare. This was cited as a problem also because at times healthcare workers were not clear about who should complete and sign J88 forms:

‘The patient had come in following having been assaulted by an adult [*patients was 8 years old*]. The family of the patient wanted a J88 filled so as to lay charges. The attending doctor had me fill out the form and sign – when I signed, then the attending doctor countersigned the document.’ (Participant 59, Rotation 2, Facility 3)

Inadequate monitoring and suboptimal management of patients in the labour wards were perceived as a cause for concern by a majority of the participants. Perceived significant events were recorded of women giving birth unattended and sometimes in corridors mostly because of delayed allocation of beds or poor monitoring:

‘In the labour ward during one of my weeknight calls a gravida 1, para 2 who had arrived in the morning was sitting on the bench in the admission cubicle as no bed had yet been allocated to her despite abundant availability of beds at that time. At about 20h00 she began shouting for a sister, saying that her baby was coming. The sister in charge of her came eventually, ambled to her cubicle, and discovered that the baby was crowning significantly. She shouted at the woman to get on the bed and then left immediately to get a delivery pack. While she was gone the baby was delivered and this occurred quickly, while the woman was still struggling to climb onto the bed.’ (Participant 88, Rotation 1, Facility 11)

## Discussion of results

This study is the first of its kind to describe the type of significant events that were observed and witnessed by medical students in PHC settings in South Africa. In related studies, medical students were based in high-income countries in academic teaching hospitals in Australia^15^ and in Connecticut, USA.^16^ In this study, medication and prescription errors were common during the prescribing phase and were often discovered later when patients presented for review dosages and inappropriate frequencies. Data on medication error prevalence are not readily available in South Africa, and according to an article by Truter et al.,^38^ although medication errors are prevalent in South Africa, they are understudied. This supports views reported by Cox and Holden^39^ and, while their data are somewhat dated, not only did they highlight the frequency of medication errors, but also they identified harms that accrue to patients. Notwithstanding similarities in our findings to those reported by Truter et al.,^38^ their study was based in an academic teaching hospital and reported on observations made about experiences of paediatric patients who were particularly prone to medical errors that included incorrect dosing, omission of medication and medication given at the incorrect time. Arguably, some of the harms were perceived to be preventable, indicating opportunities for improving patient safety. Notably, one-third of the medical errors were associated with groups of medications such as anti-infectives and analgesics, whereas in our study, errors were commonly identified in the management of human resource virus (HIV) and tuberculosis (TB), underlining the significance of managing the risks and challenges related to these chronic conditions in South Africa. In addition to the aforementioned, this study also established that a few healthcare workers seldom consider the contraindications of medications when prescribing, especially in patients with chronic conditions. In a study by Marchon, Mendes and Pavão,^40^ the authors reported that when staff fail to observe drug contraindications, this is harmful to patients. A significant concern in South Africa is that PHC facilities are the initial point of contact between a patient and the health system in addition to being a conduit for chronic patients to access life-preserving medication. As medication and prescription errors are often discovered when a patient presents for their review, this not only delays the recovery of patients but also adds a burden to an already strained health system. Overall, these findings underscore the importance of medication safety protocols, effective drug monitoring and healthcare provider education to minimise the occurrence of preventable medication errors.

Another significant event according to the participants was diagnostic errors. The most common diagnostic errors mentioned in this study included missed and wrong diagnoses.^41^ Diagnostic errors are considered a major challenge as they can lead to prescribing errors, which in turn delay treatment of the real condition. As medication and prescription errors are an outcome of a complex system, reviewing failures within the health system could assist healthcare workers in addressing such problems and thus improve patient safety. Steinhardt et al.^40^ state that disease diagnosis is a challenge in PHC facilities, particularly in low- and middle-income countries, because of unavailability of essential diagnostic tools. As a result, healthcare workers use clinical signs and symptoms to treat patients. Consequently, Reid et al.^41^ and Visagie and Schneider^42^ argue that healthcare workers in PHC facilities are competent in managing common presenting conditions they are familiar with in primary level settings. They may not always have the capacity to manage complex medical conditions, especially when they do not receive adequate support from the secondary levels of care concurring with study findings. Singh et al.^43^ state that diagnostic errors occur when healthcare workers do not have adequate time to make clinical decisions because of high workloads. These findings highlight the need for improved diagnostic capabilities in PHC facilities, especially in resource-limited settings. Additionally, enhancing support and communication between primary and secondary levels of care could help reduce diagnostic errors and improve patient outcomes. Addressing workload concerns and providing adequate time for clinical decision-making are also crucial to mitigate the occurrence of diagnostic errors.

In this study, inadequate patient management, a concern shared by Dapaah,^44^ resulted in the physical and verbal abuse of patients, particularly in the labour wards, as well as poor management of rape and assault cases.^45^ These findings correspond with those reported by Marchon et al.,^45^ as they argue that healthcare workers do not always conduct a comprehensive physical examination and therefore fail to diagnose and treat patients adequately. According to Zitha and Mokgatle,^46^ women abuse in maternity wards is a concern for healthcare facilities globally. Gravely concerned about the interactions between patient and healthcare workers, Dapaah^44^ observed that healthcare workers in sub-Saharan Africa were often harsh to patients. In their study, Jina et al.^47^ reported that South African healthcare workers in different provinces had little knowledge of working with post-rape cases despite participation in sensitivity training programmes. They further argued that there is a correlation between healthcare worker knowledge and attitudes in delivering sensitive services to patients.^47^ The poor treatment of some of the patients in PHC facilities highlighted the lack of patient-centred care, which not only compromises the safety of patients but may also cause poor health-seeking behaviours among patients. The findings show the need for comprehensive and patient-centred approaches in patient management within PHC facilities. Proper training and support for healthcare workers dealing with sensitive cases, such as caring for post-rape survivors, is crucial. By improving healthcare workers’ knowledge and attitudes, as well as promoting patient-centred care, healthcare facilities can better serve their patients and enhance patient safety and overall health outcomes.

In this study, poor clinical records resulted in poor decision-making in the diagnosis, treatment, management and prescription of medication to patients. According to Mathioudakis et al.,^48^ keeping clinical records is necessary to ensure patients receive adequate quality care, as it ensures continuity of care and improved communication between different healthcare professionals. Shihundla et al.^49^ argue that in South Africa, PHC workers, in particular nurses, have very high workloads and sometimes cannot maintain good clinical records supporting the observations of the study participants. As such, poor clinical record keeping presented a challenge in PHC facilities resulting in poor patient care.

### Limitations of the study

Significant event analysis data used in the study were based on medical students’ recordings of what they perceived as significant events. There was no objective validation of these events. Triangulation using students’ reflections and a review of significant events reported in the healthcare facilities would have strengthened the study. Data employed in this study were drawn from reflections of the 2014 final year class and therefore open to time bias.

## Conclusion

In conclusion, this study highlights the pressing challenges faced by South Africa’s PHC facilities in instituting effective management of significant events. The findings underscore the critical need for improving medication safety protocols, enhancing diagnostic capabilities and providing adequate support and training of healthcare workers. Addressing these issues can lead to better patient outcomes, reduce medical errors and implement a more patient-centred and efficient healthcare system. Additionally, emphasising the importance of maintaining accurate clinical records can ensure continuity of healthcare and effective communication among healthcare professionals. Collaborative efforts among stakeholders and policymakers are essential to bring about the necessary improvements in the country’s PHC settings.

### Contribution

The study demonstrated medical students’ ability to identify incidents in the care of patients using the SEA approach and their role in assessing patient safety issues in PHC settings.
